# Publisher Correction: ER stress-induced mediator C/EBP homologous protein thwarts effector T cell activity in tumors through T-bet repression

**DOI:** 10.1038/s41467-019-11563-5

**Published:** 2019-08-15

**Authors:** Yu Cao, Jimena Trillo-Tinoco, Rosa A. Sierra, Carmen Anadon, Wenjie Dai, Eslam Mohamed, Ling Cen, Tara L. Costich, Anthony Magliocco, Douglas Marchion, Richard Klar, Sven Michel, Frank Jaschinski, Richard R. Reich, Shikhar Mehrotra, Juan R. Cubillos-Ruiz, David H. Munn, Jose R. Conejo-Garcia, Paulo C. Rodriguez

**Affiliations:** 1 Department of Immunology, H. Lee Moffitt Cancer Center & Research Institute, Tampa, FL 33612 USA; 2 Cancer Informatics Core, H. Lee Moffitt Cancer Center & Research Institute, Tampa, FL 33612 USA; 3 Department of Pathology, H. Lee Moffitt Cancer Center & Research Institute, Tampa, FL 33612 USA; 4 Secarna Pharmaceuticals GmbH & Co. KG, 82152 Planegg/Martinsried, Germany; 5 Biostatistics Program, H. Lee Moffitt Cancer Center & Research Institute, Tampa, FL 33612 USA; 6 Department of Surgery, Medical University of South Carolina, Charleston, SC 29425 USA; 7 Department of Obstetrics and Gynecology, Weill Cornell Medicine, New York, NY 10065 USA; 8 Sandra and Edward Meyer Cancer Center, Weill Cornell Medicine, New York, NY 10065 USA; 9 Department of Pediatrics, Georgia Cancer Center, Augusta University, Augusta, GA 30912 USA

**Keywords:** Tumour immunology, Immunosurveillance

Correction to: *Nature Communications* 10.1038/s41467-019-09263-1, published online 20 March 2019.

The original version of this Article contained an error in Fig. 2. In Fig. 2d, the western blot panel relative to the human T cells (right) was an inadvertent copy of the western blot panel relative to the mouse T cells (left). This has been corrected in both the PDF and HTML versions of the Article.

