# Supplementary material for: ER stress-induced mediator C/EBP homologous protein thwarts effector T cell activity in tumors through T-bet repression
Source: Nat Commun. 2019 Mar 20;10:1280. doi: 10.1038/s41467-019-09263-1 (PMC6426975; doi:10.1038/s41467-019-09263-1)
Supplement: Supplementary file 1 — Supplementary Information [file 41467_2019_9263_MOESM1_ESM.pdf]

# SUPPLEMENTARY INFORMATION

## ER stress-induced mediator C/EBP homologous protein thwarts effector T-cell activity in tumors through T-bet repression

Cao et al.

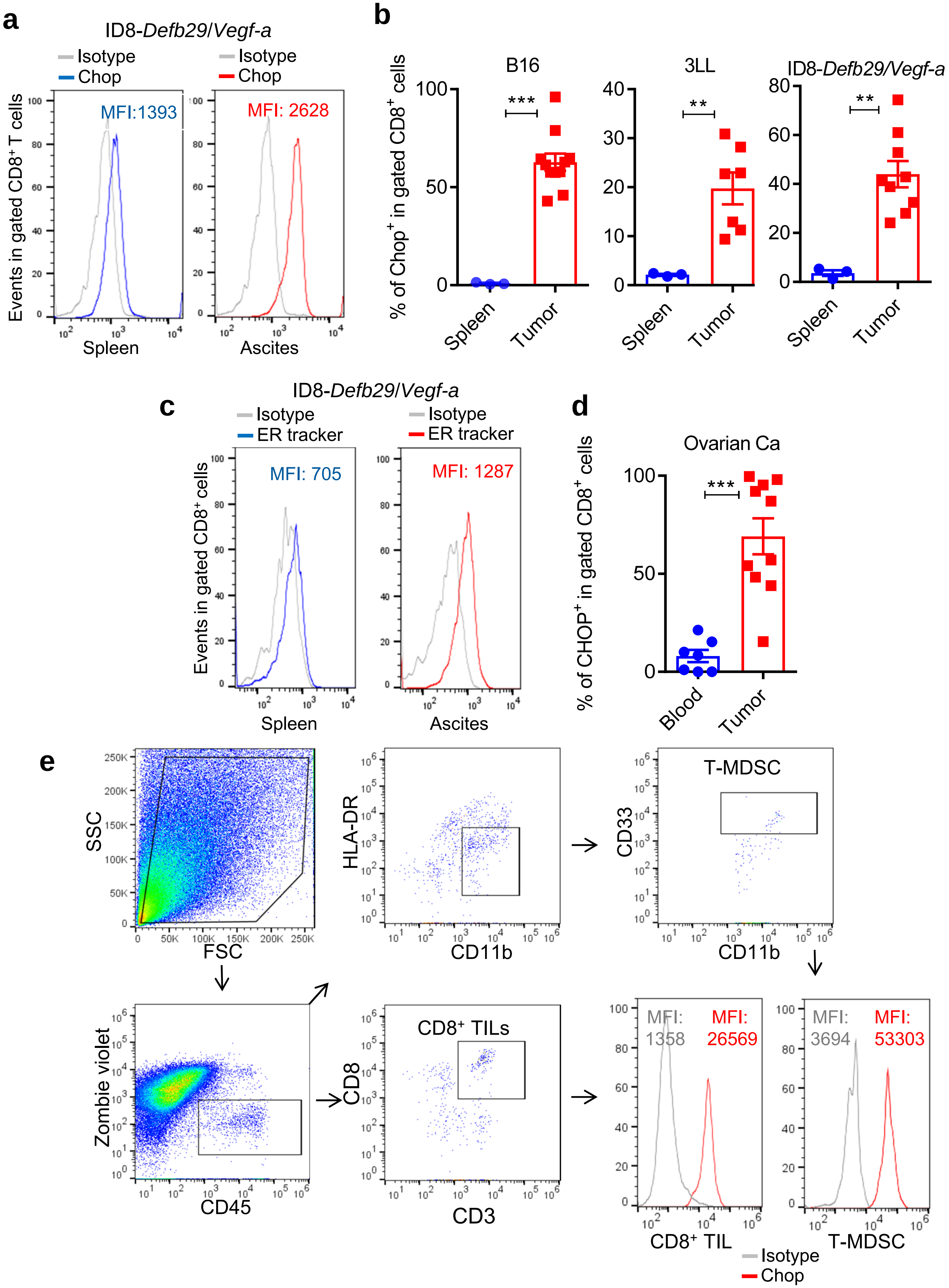
a
ID8-Defb29/Vegf-a
Isotype
Chop
Isotype
Chop
MFI:1393
MFI: 2628
Events in gated CD8⁺ T cells
Spleen
Ascites
b
% of Chop⁺ in gated CD8⁺ cells
B16
3LL
ID8-Defb29/Vegf-a
***
**
**
Spleen
Tumor
c
ID8-Defb29/Vegf-a
Isotype
ER tracker
Isotype
ER tracker
MFI: 705
MFI: 1287
Events in gated CD8⁺ cells
Spleen
Ascites
d
Ovarian Ca
% of CHOP⁺ in gated CD8⁺ cells
***
Blood
Tumor
e
SSC
FSC
HLA-DR
CD11b
T-MDSC
CD33
CD11b
Zombie violet
CD45
CD8
CD3
CD8⁺ TILs
MFI: 1358
MFI: 26569
MFI: 3694
MFI: 53303
CD8⁺ TIL
T-MDSC
Isotype
Chop

**Supplementary Figure 1. Chop is upregulated in MDSCs and CD8$^+$ TILs from murine and human ovarian tumors. a**. Chop in splenic CD8$^+$ T cells (*left*, n=3) and ascites CD8$^+$ TILs (*right*, n=9) from ID8-*Defb29*/*Vegf-a* ovarian tumor-bearing mice. Chop was detected by FACS and levels indicated by mean fluorescent intensity (MFI). **b**. Percentage of Chop$^+$ cells was established by FACS in gated splenic and tumor-associated CD8$^+$ T cells from mice bearing: *left*: B16 tumors (n=11); *middle*: 3LL tumors (n=7); *right*: ID8-*Defb29*/*Vegf-a* ovarian tumors (n=9). **c**. ER tracker in CD8$^+$ TILs from ID8-*Defb29*/*Vegf-a* ovarian tumor ascites, compared with splenic CD8$^+$ T cells from the corresponding tumor-bearing mice (n=3). ER tracker was detected by FACS and levels indicated by MFI. **d**. Percentage of Chop$^+$ cells by FACS in tumor-associated CD8$^+$ T cells from human ovarian carcinoma tissues, compared with matching peripheral blood CD8$^+$ T cells from ovarian cancer patients. Bar graphs show mean ± s.e.m. **e**. Gating strategy for tumor-infiltrating MDSC (T-MDSC, CD45$^+$ CD11b$^+$ MHC-DR$^-$ CD33$^+$) and CD8$^+$ TILs (CD45$^+$ CD3$^+$ CD8$^+$) from human ovarian carcinoma samples. CHOP levels in T-MDSC *vs*. CD8$^+$ TILs (lower *right*). CHOP expression was detected by FACS and levels indicated by MFI. Representative results from 7 independent patients. In all figures, the bar graphs show the mean ± s.e.m. *$p < 0.05$, **$p < 0.01$, ***$p < 0.001$ calculated using two-tailed unpaired Student's *t*-test.

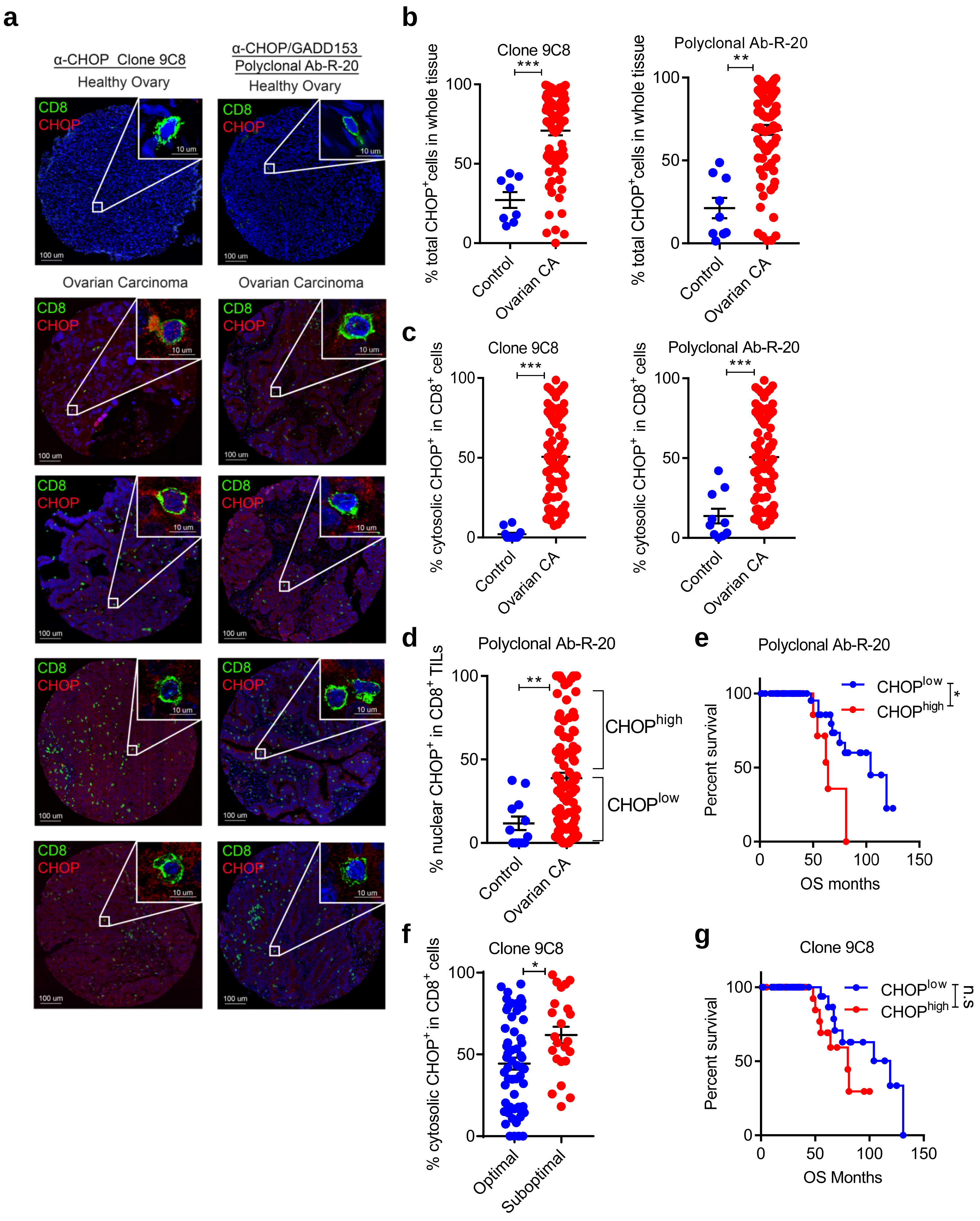

a
α-CHOP Clone 9C8
Healthy Ovary
α-CHOP/GADD153
Polyclonal Ab-R-20
Healthy Ovary
Ovarian Carcinoma
Ovarian Carcinoma
CD8
CHOP
10 um
100 um
b
Clone 9C8
Polyclonal Ab-R-20
% total CHOP+cells in whole tissue
Control
Ovarian CA
c
Clone 9C8
Polyclonal Ab-R-20
% cytosolic CHOP+ in CD8+ cells
d
Polyclonal Ab-R-20
% nuclear CHOP+ in CD8+ TILs
CHOPhigh
CHOPlow
e
Polyclonal Ab-R-20
Percent survival
OS months
f
Clone 9C8
% cytosolic CHOP+ in CD8+ cells
Optimal
Suboptimal
g
Clone 9C8
Percent survival
OS Months
n.s

**Supplementary Figure 2. CHOP is upregulated in CD8$^+$ TILs from ovarian carcinoma patients. a**. Representative panel illustrating a spectrum of CHOP expression patterns in CD8$^+$ TILs from ovarian carcinoma tissues compared to those from healthy ovary tissues. CHOP (red, clone 9C8 (*left*) or polyclonal R-20 (*right*)), CD8 (green) and DAPI (blue) were detected by confocal microscopy. **b**. Percentage of total CHOP$^+$ cells (*left*: clone 9C8; *right*: polyclonal Ab-R-20) by non-subjective imaging analysis in a tissue microarray containing ovarian carcinoma tumors and healthy ovary tissues. The graphs show the mean ± s.e.m.. **c**. Non-subjective results showing a higher frequency of CD8$^+$ T cells having cytosolic CHOP in the ovarian carcinoma tumors, compared to those from normal ovarian tissues (*left*: clone 9C8; *right*: polyclonal Ab-R-20). **d**. Percentage of nuclear-CHOP$^+$ cells (polyclonal Ab-R-20) in tumor-associated CD8$^+$ T cells from ovarian carcinoma tissues vs. healthy tissues as in **Fig. 1f**. Graph shows mean ± s.e.m.. **e**. Overall survival of ovarian tumor patients having high frequency of nuclear-CHOP in CD8$^+$ TILs (CHOP$^{high}$) (n=18) vs. low frequency of nuclear-CHOP in CD8$^+$ TILs (nuclear-CHOP$^{low}$) (n=57) (logrank 4.55, p=0.0329 using the Gehan-Breslow-Wilcoxon test; cutoff was established as in the Methods). Studies were developed using the anti-Chop polyclonal Ab-R-20. **f**. Percentage of CD8$^+$ TILs having cytosolic-CHOP (clone 9C8) in ovarian cancer patients that had optimal (n=58) vs. suboptimal (n=23) cytoreductive debulking surgery. **g**. Overall survival in ovarian tumor patients showing high frequency of cytosolic-CHOP in CD8$^+$ TILs (CHOP$^{high}$) (n=37) vs. those having low frequency of cytosolic-CHOP in CD8$^+$ TILs (CHOP$^{low}$) (n=44). CHOP was detected using the anti-Chop 9C8 clone antibody. In the bar graphs showing mean ± s.e.m., *p < 0.05, **p < 0.01, ***p < 0.001 were calculated using two-tailed unpaired Student's t-test.

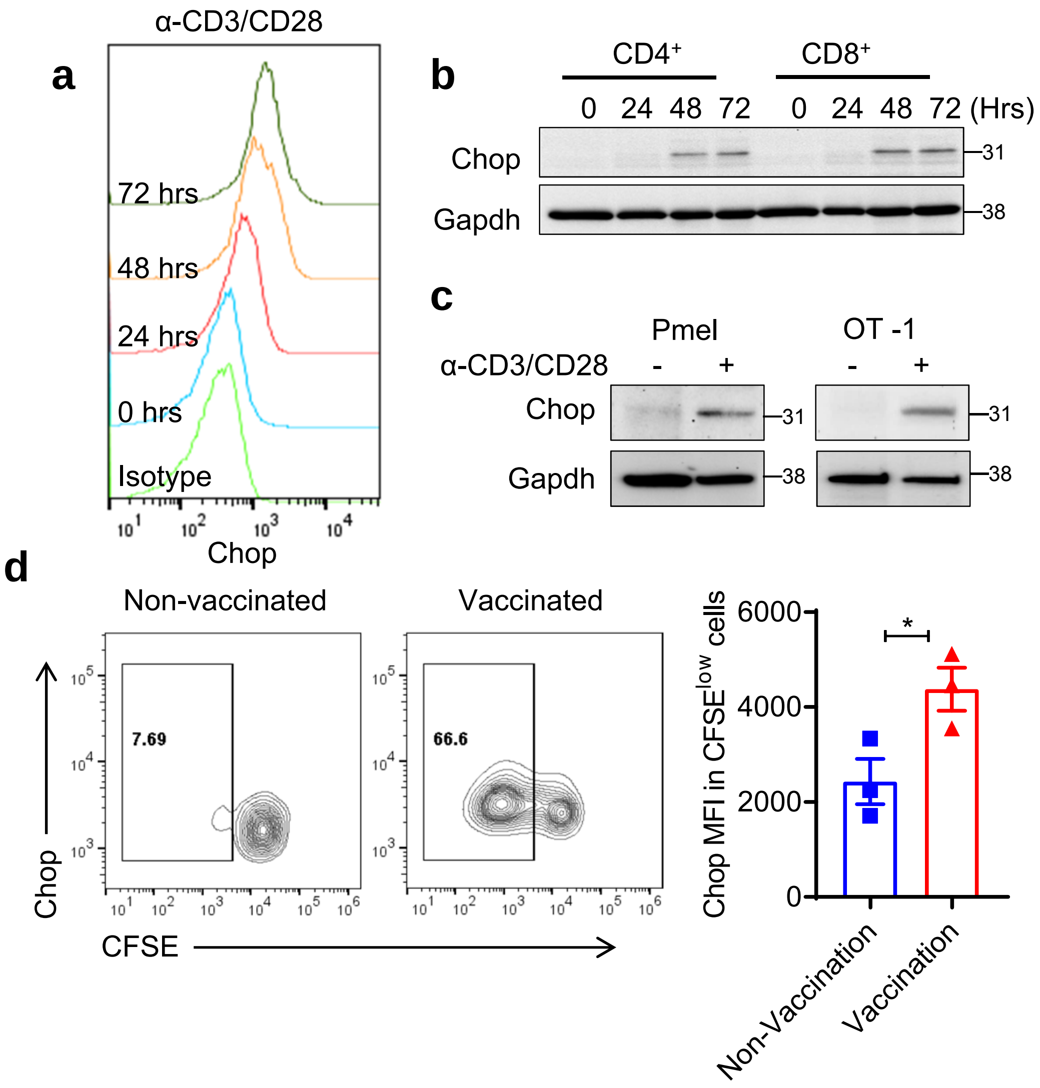


**Supplementary Figure 3. Chop is upregulated in stimulated CD8$^+$ T cells**. **a**. Time-dependent induction of Chop by FACS after CD8$^+$ T cell priming with anti-CD3/CD28 (0-72 hours; n=4). **b**. Upregulation of Chop in anti-CD3/CD28-primed CD4$^+$ or CD8$^+$ T cells (0-72 hours; n=3). **c**. Chop levels in Pmel and OT-1 CD8$^+$ T cells stimulated for 48 hours with gp100$_{25\text{-}33}$ or OVA$_{257\text{-}264}$, respectively. (n=3). **d**. Chop expression was assessed by FACS in proliferating transferred Pmel T-cells from gp100$_{25\text{-}33}$-vaccinated mice (activation driven T cell proliferation) compared to non-vaccinated cohorts (homeostatic T cell division). *Left*: representative gating of CSFE$^{diluted}$ proliferating cells; *right*: Bar graph showing mean ± s.e.m. of Chop levels (n=3) in gated CD8$^+$ CD90.1$^+$ CSFE$^{diluted}$ proliferating T cells. *p < 0.05 by two-tailed unpaired Student’s *t*-test.

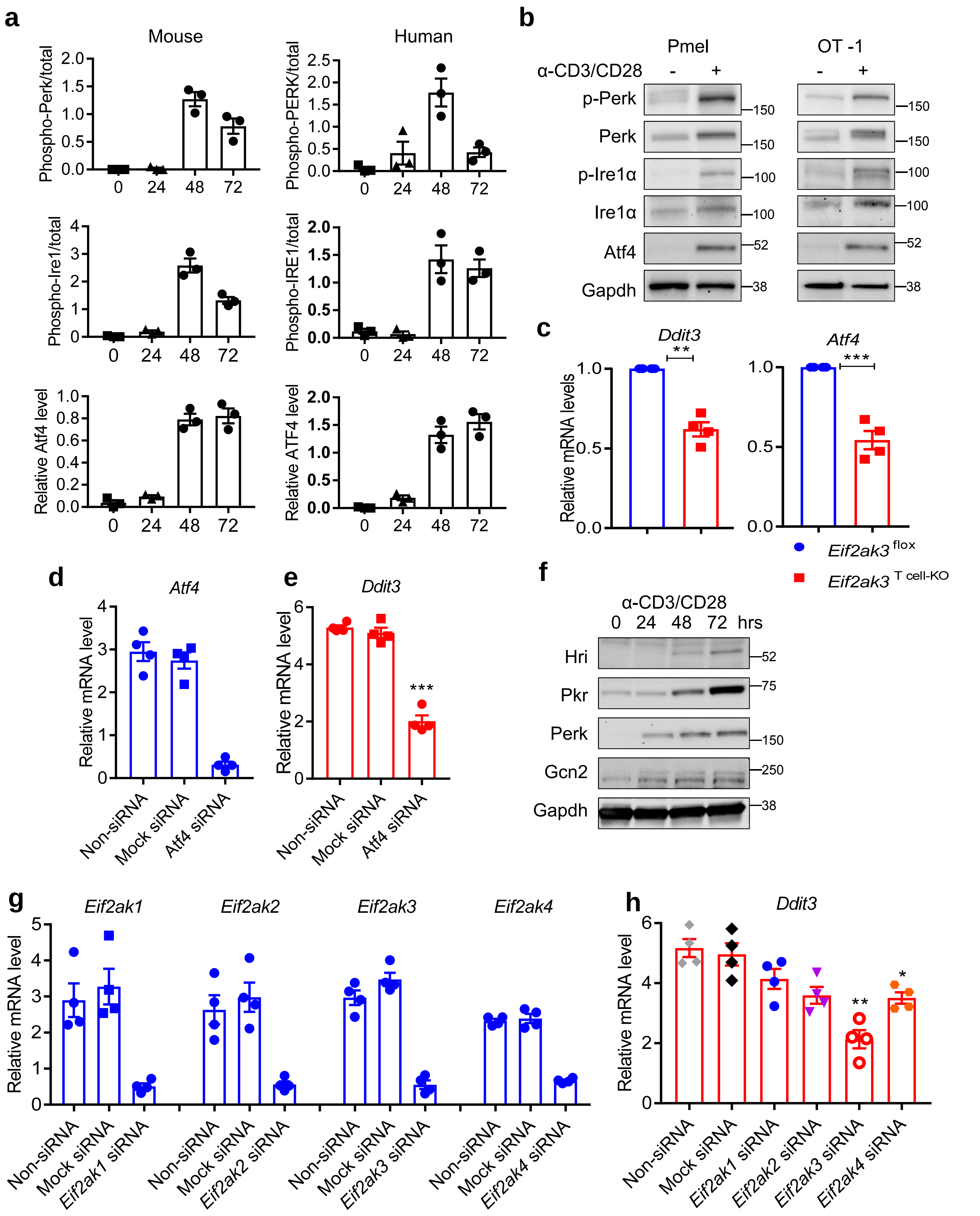

a
Mouse
Human
Phospho-Perk/total
Phospho-PERK/total
Phospho-Ire1/total
Phospho-IRE1/total
Relative Atf4 level
Relative ATF4 level
0 24 48 72
b
Pmel
OT -1
α-CD3/CD28
p-Perk
Perk
p-Ire1α
Ire1α
Atf4
Gapdh
c
Ddit3
Atf4
Relative mRNA levels
Eif2ak3 flox
Eif2ak3 T cell-KO
d
Atf4
e
Ddit3
Relative mRNA level
Non-siRNA
Mock siRNA
Atf4 siRNA
f
α-CD3/CD28
0 24 48 72 hrs
Hri
Pkr
Perk
Gcn2
Gapdh
g
Eif2ak1
Eif2ak2
Eif2ak3
Eif2ak4
Eif2ak1 siRNA
Eif2ak2 siRNA
Eif2ak3 siRNA
Eif2ak4 siRNA
h
Ddit3

**Supplementary Figure 4. Chop induction in activated CD8$^+$ T cells depends on the ER stress-associated kinase Perk**. **a**. Densitometry quantitation of immunoblots from **Fig. 2d** and that merges the results from 3 repeats. **b**. UPR mediators in Pmel and OT-1 CD8$^+$ T cells stimulated for 48 hours with gp100$_{25\text{-}33}$ or OVA$_{257\text{-}264}$, respectively. (n=3). **c**. *Ddit3* and *Atf4* mRNA levels in stimulated *Eif2ak3*-null and *Eif2ak3*-flox CD8$^+$ T cells. (n=4). **d**. *Atf4* mRNA levels in stimulated CD8$^+$ T cells transfected with non-targeting siRNA or a pool of specific *Atf4* siRNA. Results are from 4 independent repeats. **e**. *Ddit3* mRNA levels in stimulated CD8$^+$ T cells transfected as in (**d**). **f**. Hri, Pkr, Perk, and Gcn2 levels in anti-CD3/CD28-stimulated murine CD8$^+$ T cells (0-72 hours). Results are a representative experiment from 3 repeats. **g**. siRNA-based silencing of *Eif2ak1*, *Eif2ak2*, *Eif2ak3*, and *Eif2ak4* upon CD8$^+$ T cell priming. Results are from 4 repeats. **h**. *Ddit3* mRNA levels in stimulated CD8$^+$ T cells from (**g**). All the bar graphs show mean ± s.e.m.. *$p < 0.05$, **$p < 0.01$, ***$p < 0.001$ were calculated using two-tailed unpaired Student's *t*-test.

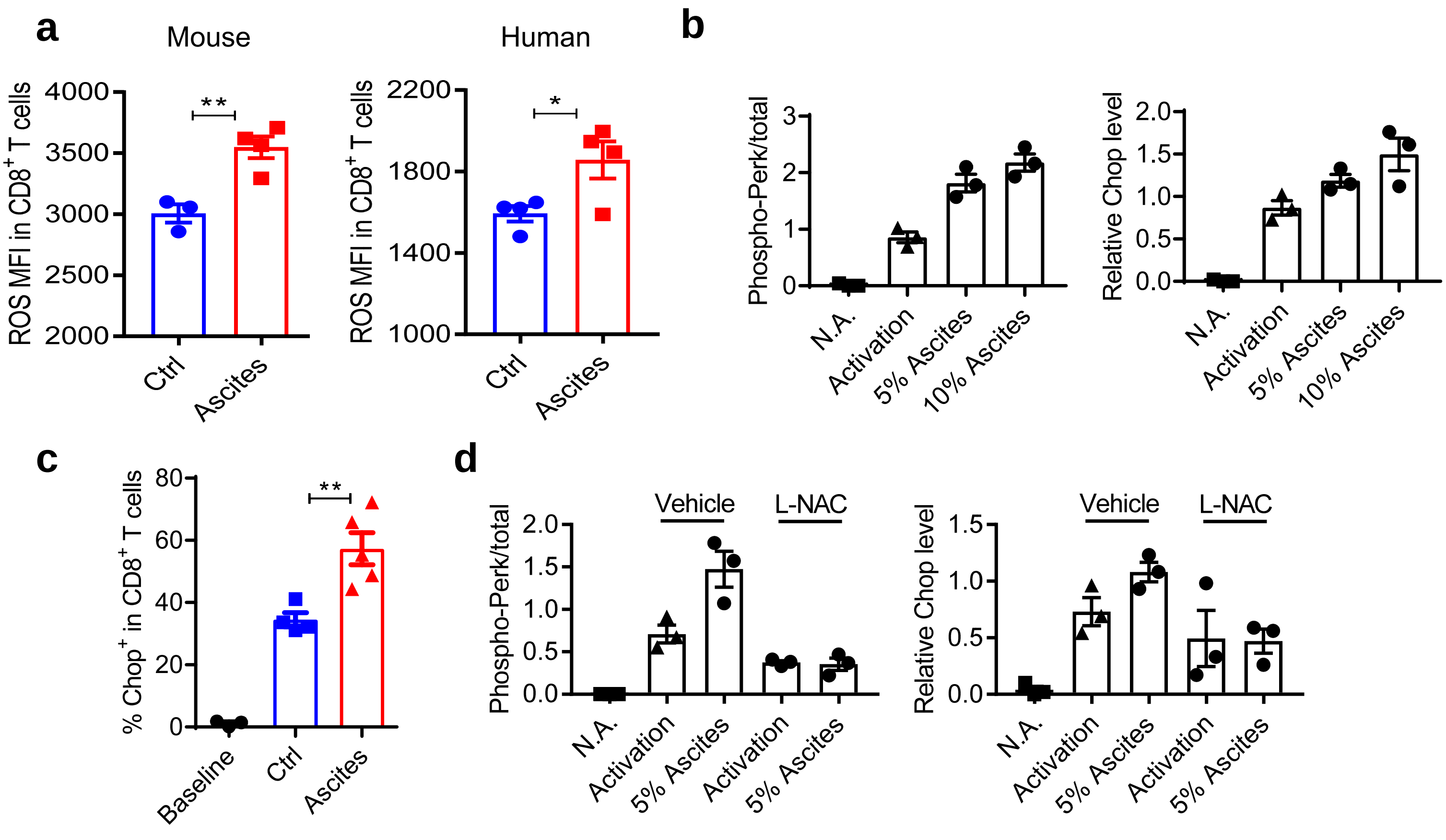


**Supplementary Figure 5. ROS-induced Perk activation regulates Chop induction in tumor-exposed CD8+ T-cells. a**. ROS levels were monitored by FACS in mouse and human DHE-labeled CD8+ T-cells activated with anti-CD3/CD28 and treated for 24 hours with 5% cell-free ovarian ascites from mice bearing ID8-*Defb29*/*Vegf-a* ovarian tumors, or 5% primary ascites from patients with ovarian cancer, respectively. (n=4). **b**. Merged densitometry quantitation of immunoblots from **Fig. 2h**. Results are from 3 independent repeats. **c**. Percentage of Chop+ cells by FACS in primed CD8+ T cells treated with 5% cell-free ID8-*Defb29*/*Vegf-a* ovarian tumor ascites for 24 hours, compared with vehicle treated CD8+ T cells. (n=5). **d**. Densitometry quantitation of immunoblot intensities from **Fig. 2i**. Results are from 3 independent repeats. All bar graphs show the mean ± s.e.m. *p < 0.05, **p < 0.01 were calculated using two-tailed unpaired Student’s *t*-test.

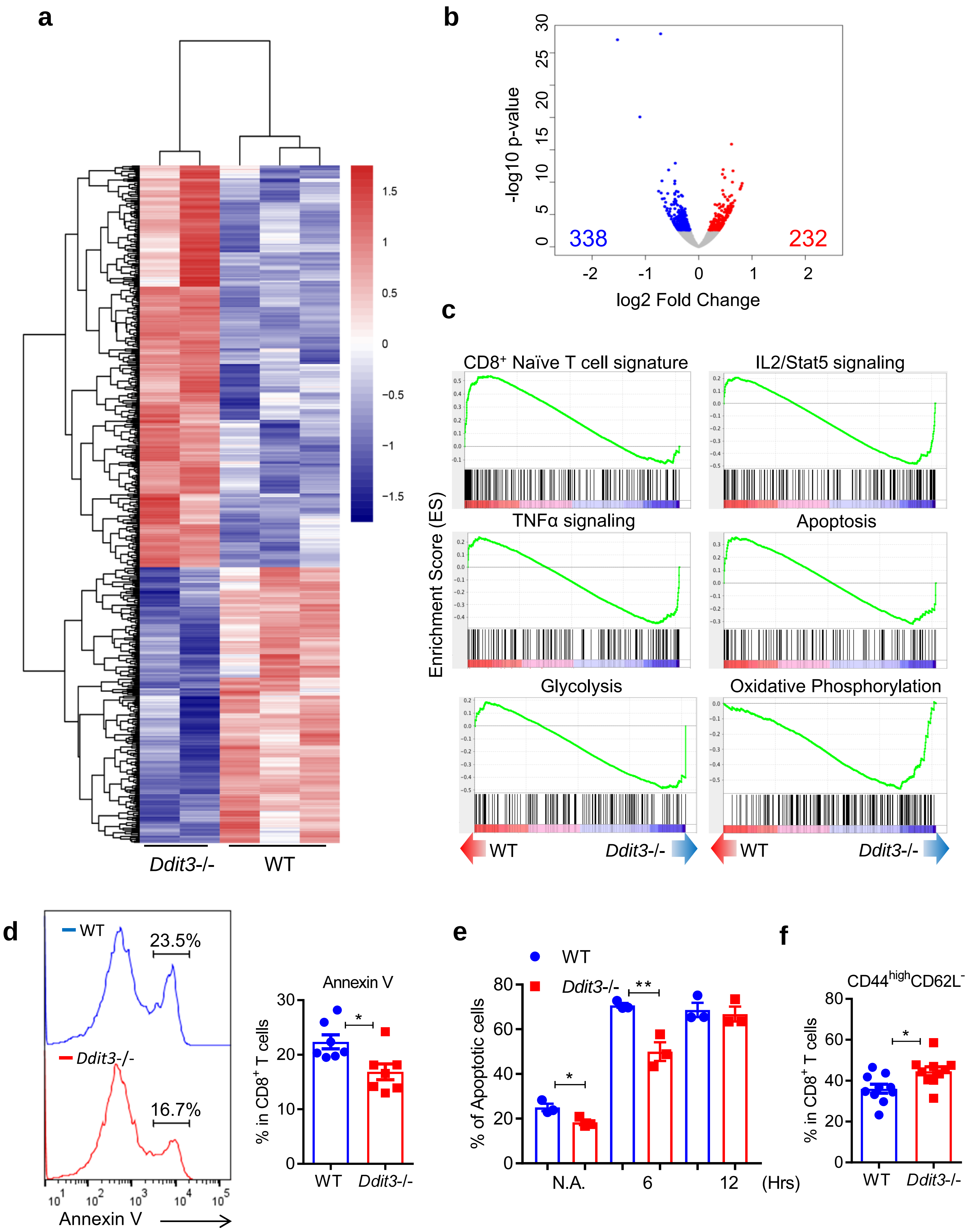
a
1.5
1
0.5
0
−0.5
−1
−1.5
Ddit3-/-
WT
b
-log10 p-value
log2 Fold Change
338
232
c
Enrichment Score (ES)
CD8+ Naïve T cell signature
IL2/Stat5 signaling
TNFα signaling
Apoptosis
Glycolysis
Oxidative Phosphorylation
WT
Ddit3-/-
d
WT
23.5%
Ddit3-/-
16.7%
Annexin V
% in CD8+ T cells
e
WT
Ddit3-/-
% of Apoptotic cells
N.A.
6
12
(Hrs)
f
CD44highCD62L-
% in CD8+ T cells

**Supplementary Figure 6. Chop plays a primary role in $CD8^+$ T cell function. a-b**. Heatmap (**a**) and plot distribution (**b**) from RNA-Seq detection of activated wild-type (n=3) *vs*. *Ddit3*-/- (n=2) Pmel $CD8^+$ T cells. **c**. GSEA of specific gene signatures in primed wild-type vs. *Ddit3*-/- Pmel $CD8^+$ T cells. **d**. Spontaneous frequency of Annexin $V^+$ cells in wild-type and *Ddit3*-/- $CD8^+$ T cells activated in the presence of anti-CD3/CD28 for 72 hours. *Left*: representative result; *right*: mean ± s.e.m. from 7 repeats. **e**. Annexin $V^+$ cells in wild-type or *Ddit3*-/- $CD8^+$ T cells primed *in vitro* for 48 hours and then treated with 1µM staurosporine for additional 6 or 12 hours. (n=3). **f**. Merged data from **Fig. 3k** showing the frequency of $CD44^{high}$ $CD62L^-$ cells within wild-type or *Ddit3*-null $CD8^+$ T-cells. (n=10). $^*p < 0.05$, $^{**}p < 0.01$were calculated using two-tailed unpaired Student's *t*-test.

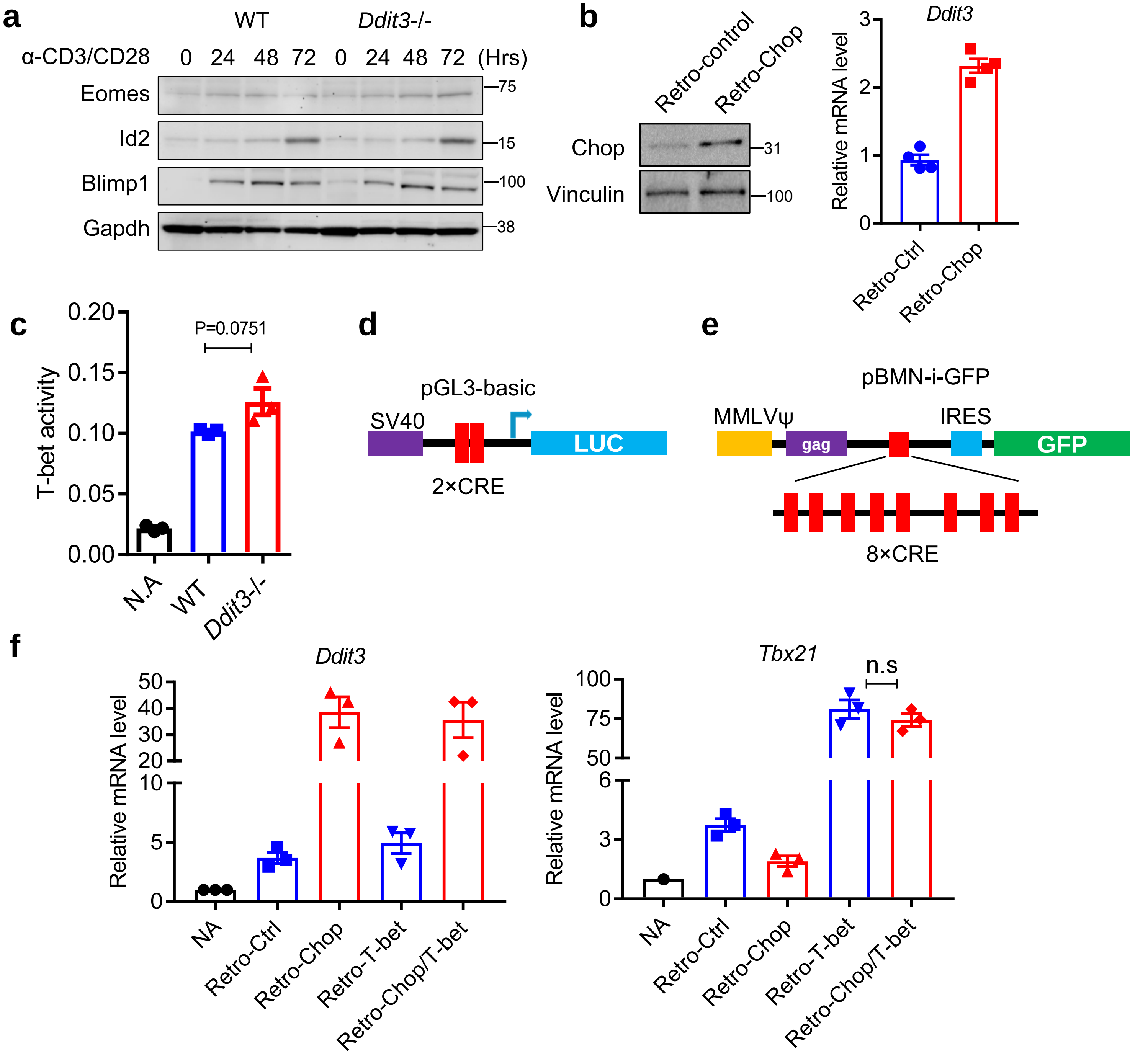


**Supplementary Figure 7. Mechanistic and functional interaction between Chop and T-bet in activated CD8$^+$ T cells. a**. Time-course expression of Eomes, Id2, and Blimp1 in wild-type and *Ddit3*-/- CD8$^+$ T-cells stimulated for 72 hours with plate bound anti-CD3/CD28. (n=3). **b**. Expression of Chop protein (*left*, short-time exposure) and *Ddit3* mRNA (*right*) in primed CD8$^+$ T cells infected with control (Retro-ctrl) or *Ddit3*-coding retrovirus (Retro-Chop). (n=3). **c**. DNA-binding activity (TransAM) of T-bet in primed wild-type or *Ddit3*-/- CD8$^+$ T cells. **d**. Model of SV40-2CRE-Luc system. **e**. Model of MSCV-8CRE-GFP system. **f**. *Ddit3* and *Tbx21* mRNA in primed CD8$^+$ T cells transduced with: 1) CD90.1 and GFP expressing control virus (Retro-Ctrl); 2) *Ddit3*/CD90.1 expressing virus and GFP expressing control virus (Retro-Chop); 3) CD90.1 expressing control virus and *Tbx21*/GFP expressing virus (Retro-T-bet); or 4) Chop/CD90.1 expressing virus and T-bet/GFP expressing virus (Retro-Chop/T-bet). Bar graphs show the mean ± s.e.m. (n=3). *p < 0.05, **p < 0.01, ***p < 0.001 calculated using two-tailed unpaired Student's *t*-test.

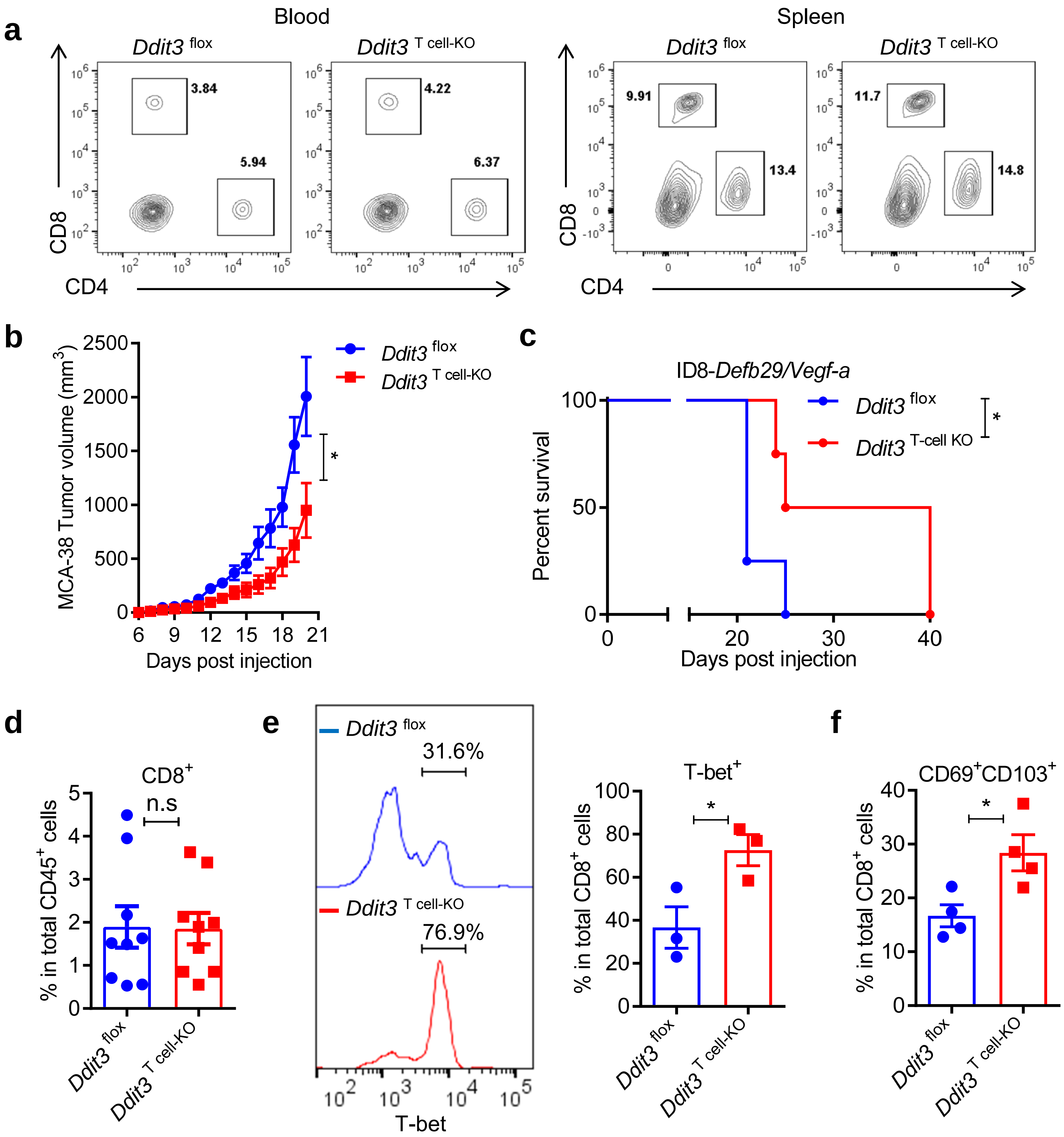


**Supplementary Figure 8. Chop intrinsically regulates anti-tumor T cell immunity**. **a**. Distribution of CD4$^+$ and CD8$^+$ T cells in spleen and peripheral blood from *Ddit3*$^{flox}$ and *Ddit3*$^{T\ cell\text{-}KO}$ mice. (n=5). **b**. Tumor growth in *Ddit3*$^{flox}$ (blue) or *Ddit3*$^{T\ cell\text{-}KO}$ (red) mice bearing MCA-38 tumors. Average kinetics ± s.e.m. of 6 mice/group. **c**. Survival of *Ddit3*$^{flox}$ or *Ddit3*$^{T\ cell\text{-}KO}$ mice bearing ID8-*Defb29*/*Vegf-a* ovarian tumors. (n=4; logrank 4.14, p=0.046 using the Gehan-Breslow-Wilcoxon test). **d**. Frequency of CD8$^+$ TILs from B16 tumors collected from *Ddit3*$^{flox}$ or *Ddit3*$^{T\ cell\text{-}KO}$ mice. (n= 9 mice/group). **e**. T-bet levels in CD8$^+$ TILs from *Ddit3*$^{flox}$ or *Ddit3*$^{T\ cell\text{-}KO}$ mice bearing s.c. B16 tumors. *Left*: representative result; *right*: mean ± s.e.m. from 3 mice. **f**. Percentage of CD69$^+$ CD103$^+$ resident memory CD8$^+$ TILs in B16 tumors isolated from *Ddit3*$^{flox}$ or *Ddit3*$^{T\ cell\text{-}KO}$ mice. (n=4). In the bar graphs, *p < 0.05, **p < 0.01, ***p < 0.001 were calculated using two-tailed unpaired Student's *t*-test.

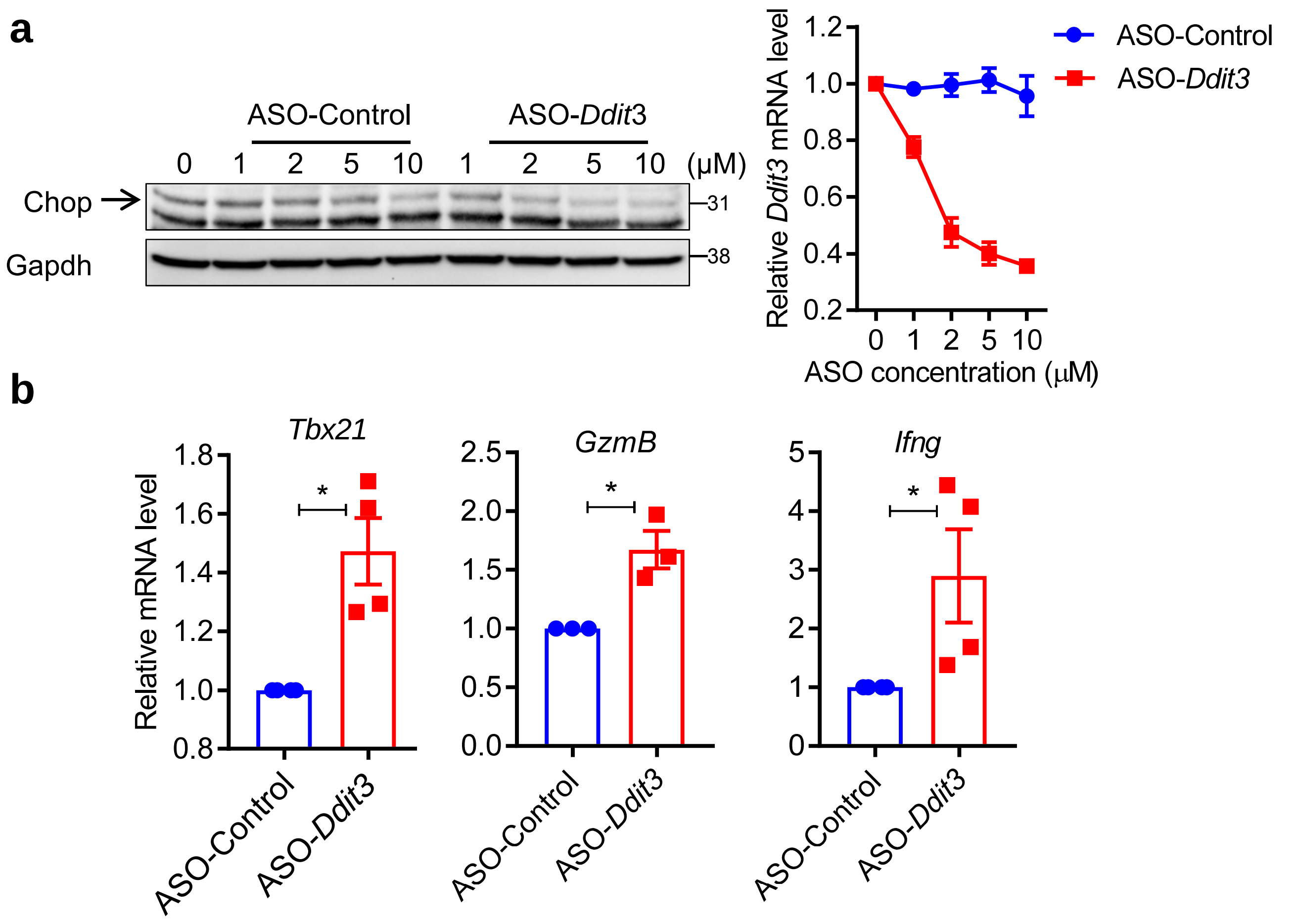


**Supplementary Figure 9. Targeted silencing of Chop in CD8⁺ T cells modulates expression of effector molecules. a**. Inhibited Chop expression in primed T cells treated with ASO-Control or ASO-*Ddit3*. *Left*: Representative experiment showing Chop protein levels (n=3); *right*: *Ddit3* mRNA from 3 independent studies. **b**. *Tbx21, Gzmb and Ifng* mRNA levels (mean ± s.e.m.) in primed Pmel T cells treated with ASO-Control or ASO-*Ddit3*. (n=4 for *Tbx21 and Ifng*, n=3 for *Gzmb*, *$p < 0.05$ calculated using two-tailed unpaired Student's *t*-test).
