# Supplementary material for: ER stress-induced mediator C/EBP homologous protein thwarts effector T cell activity in tumors through T-bet repression
Source: Nat Commun. 2019 Mar 20;10:1280. doi: 10.1038/s41467-019-09263-1 (PMC6426975; doi:10.1038/s41467-019-09263-1)
Supplement: Supplementary file 3 — Reporting Summary [file 41467_2019_9263_MOESM3_ESM.pdf]

nature research

Corresponding author(s): Paulo C. Rodriguez
Last updated by author(s): Feb 4, 2019

Reporting Summary

Nature Research wishes to improve the reproducibility of the work that we publish. This form provides structure for consistency and transparency in reporting. For further information on Nature Research policies, see Authors & Referees and the Editorial Policy Checklist.

Statistics

For all statistical analyses, confirm that the following items are present in the figure legend, table legend, main text, or Methods section.

n/a	Confirmed	
☐	☒	The exact sample size (*n*) for each experimental group/condition, given as a discrete number and unit of measurement
☐	☒	A statement on whether measurements were taken from distinct samples or whether the same sample was measured repeatedly
☐	☒	The statistical test(s) used AND whether they are one- or two-sided<br>*Only common tests should be described solely by name; describe more complex techniques in the Methods section.*
☒	☐	A description of all covariates tested
☒	☐	A description of any assumptions or corrections, such as tests of normality and adjustment for multiple comparisons
☐	☒	A full description of the statistical parameters including central tendency (e.g. means) or other basic estimates (e.g. regression coefficient) AND variation (e.g. standard deviation) or associated estimates of uncertainty (e.g. confidence intervals)
☒	☐	For null hypothesis testing, the test statistic (e.g. *F*, *t*, *r*) with confidence intervals, effect sizes, degrees of freedom and *P* value noted<br>*Give P values as exact values whenever suitable.*
☒	☐	For Bayesian analysis, information on the choice of priors and Markov chain Monte Carlo settings
☒	☐	For hierarchical and complex designs, identification of the appropriate level for tests and full reporting of outcomes
☒	☐	Estimates of effect sizes (e.g. Cohen's *d*, Pearson's *r*), indicating how they were calculated

Our web collection on statistics for biologists contains articles on many of the points above.

Software and code

Policy information about availability of computer code

Data collection	No customized software was used. Applied Biosystems StepOnePlus real-time PCR system and associated software were used to collect real-time PCR data. Western blot data were collected in a ChemiDoc™ Imaging System (Bio-Rad) and densitometry analyses completed using the Image Lab™ Software (Bio Rad). FACS acquisition was performed in a CytoFLEX II (Beckman Coulter), LSRII (BD) or FACSAria II unit (BD) and analyzed using FlowJo 10.3. Samples were processed for RNA-sequencing using the NuGen Ovation Mouse RNA-Seq Multiplex System (NuGEN Technologies). The libraries were then sequenced in an Illumina NextSeq 500 v2 sequencer with 75-base single-end run. Oxygen consumption rates (OCR) and extracellular acidification rates (ECAR) were measured using a XF96 extracellular flux analyzer (Seahorse Bioscience). Immunofluorescence of the tissue microarray histology slides were visualized in a Leica SP8 Confocal microscope, and then scanned using the APERIO ScanScope FL (Leica Biosystems, Wetzlar, Germany). Images were stored in Aperio's Spectrum software, segmented into individual cores using the software's TMA lab module, and individual core images analyzed using the Definiens Tissue Studio v4.2 suite cellular analysis. Additional information about used software has been described in the manuscript or is available upon reasonable request.
Data analysis	No custom-made software was used for data analysis. TopHat v2.0.13 was used for RNA-Seq reads alignment; HTSeq v0.6.1 was used to quantify the aligned RNA-Seq reads; DESeq2 v1.6.3 was used for read counts normalization. Gsea-3.0 was used for gene set enrichment analysis. FACS data was analyzed using nalyzed using FlowJo 10.3. Graphpad Prism 7.03 was used for generating graphs and performing statistical studies.

For manuscripts utilizing custom algorithms or software that are central to the research but not yet described in published literature, software must be made available to editors/reviewers. We strongly encourage code deposition in a community repository (e.g. GitHub). See the Nature Research guidelines for submitting code & software for further information.

Data

Policy information about availability of data

All manuscripts must include a data availability statement. This statement should provide the following information, where applicable:
- Accession codes, unique identifiers, or web links for publicly available datasets
- A list of figures that have associated raw data
- A description of any restrictions on data availability

The RNA-Seq raw data that support the findings of the study have been deposited in Gene Expression Omnibus database, the GEO accession number is GSE112823. The authors declare that the all data supporting the findings of this study are available within the paper and its supplementary figures. The supporting raw data of this study are available from the corresponding author upon reasonable request.

Field-specific reporting

Please select the one below that is the best fit for your research. If you are not sure, read the appropriate sections before making your selection.

☒ Life sciences ☐ Behavioural & social sciences ☐ Ecological, evolutionary & environmental sciences

For a reference copy of the document with all sections, see nature.com/documents/nr-reporting-summary-flat.pdf

Life sciences study design

All studies must disclose on these points even when the disclosure is negative.

Sample size	Most of our experiments are in vitro experiments using primary cells from mice or donors. Sample sizes were chosen on the basis of our previous studies or publications, as well as the availability of mice and donors. For most of the in vitro studies, 3 independently developed repeats were used. Power analysis at 80% power and type I error controlled at 0.05 were used for most of the animal experiments. For simplicity, t test was used for the power analysis.
Data exclusions	We excluded one set of data from RNA-Seq dataset. Based on the PCA study, we found one sample in the Ddit3-/- group was clustered very differently from the other two replicates, we then identified it as an outlier and ruled it out from the analysis.
Replication	All attempts to reproduce the results were successful. Thus, all experiments were reliably reproduced and results are represented as mean +/- SEM or +/-SD as appropriate, which is indicated in figure legends. Unpaired Student's t-tests or paired t-tests were used to compare two independent or two paired groups, respectively.
Randomization	In the tissue micro-array studies, the ovarian cancer samples were collected randomly by Moffitt Cancer Center Tissue Core prior to this study. Commercially available buffy coats (One-blood) were used to isolate human CD8+ T cells and were from de-identified and randomly picked up healthy donors.
Blinding	The statistical studies for tissue micro-array results and RNA-Seq results have been done by Moffitt's cancer bio-informatics and statistics group in a blinded way. For the rest of the experiments, considering appropriate handling and data acquisition, investigators were not blinded to the studies. Moreover, because samples were treated equally and data collection and/or analysis were mainly performed by computer-based methods (such as flow cytometric analysis), we believe the blinding was not necessary to our study.

Reporting for specific materials, systems and methods

We require information from authors about some types of materials, experimental systems and methods used in many studies. Here, indicate whether each material, system or method listed is relevant to your study. If you are not sure if a list item applies to your research, read the appropriate section before selecting a response.

Materials & experimental systems

n/a	Involved in the study	
☐	☒	Antibodies
☐	☒	Eukaryotic cell lines
☒	☐	Palaeontology
☐	☒	Animals and other organisms
☐	☒	Human research participants
☒	☐	Clinical data

Methods

n/a	Involved in the study	
☒	☐	ChIP-seq
☐	☒	Flow cytometry
☒	☐	MRI-based neuroimaging

Antibodies

Antibodies used	Rabbit polyclonal anti-CHOP (R-20) Santa Cruz K1714<br>Mouse monoclonal anti-Chop (9C8) Abcam ab11419

Rabbit monoclonal anti-p-Perk (T980) (16F8) Cell Signaling Tech 3179S
Rabbit monoclonal anti-Perk (C33E10) Cell Signaling Tech 3192S
Rabbit monoclonal anti-IREalpha (14C10) Cell Signaling Tech 3294S
Rabbit polyclonal anti-p-IREalpha (S724) Abcam ab48187
ATF-4 (D4B8) Rabbit mAb Cell Signaling Tech 11815S
InVivo MAb anti-mouse CD8 (Lyt 2.1) BioXcell BE0118
CD8 (C8/144B) Mouse Monoclonal Antibody Cell Marque 108M-98
Mouse anti-Tbet (04-46) BD Biosciences 37776
Rabbit anti-Granzyme B Cell Signaling Tech 4275S
Anti-PKR antibody [EPR19374] Abcam ab184257
GCN2 Antibody Cell Signaling Tech 3302S
HRI Antibody (7H3L3) Invitrogen 702551
BLIMP1/PRDM1 Antibody (3H2-E8) Novus Biologicals NB600-235SS
Anti-ID2 antibody [2457C5a] Abcam Ab53545
EOMES (D8D1R) Rabbit mAb Cell Signaling Tech 81493S
Mouse monoclonal anti-GAPDH (6C5) Fitzgerald 10R-G109A
Mouse IgG1 Isotype Control, FITC eBioscience GM4992
Mouse IgG2a kappa Isotype Control, PE eBioscience 12-4724-82
Mouse IgG1 kappa Isotype Control, APC eBioscience 17-4714-42
Mouse IgG2a, kappa Isotype, Brilliant Violet 421™ Biolegend 400260
Mouse IgG2a, kappa Isotype, Brilliant Violet 785™ Biolegend 400273
Purified Mouse IgG1 Isotype Control eBioscience MG1-45
Purified Mouse IgG2b Isotype Control eBioscience MPC-11
Purified Rabbit IgG Isotype Control Cell Signaling Tech DA1E
CD8a Monoclonal Antibody (SK1), APC eBioscience 17-0087-42
CD8a Monoclonal Antibody (HIT8a), FITC eBioscience 11-0089-42
anti-mouse CD45, Brilliant Violet 785™ Biolegend 103149
CD4 Monoclonal Antibody (GK1.5), PE eBioscience 12-0041-82
CD3e Monoclonal Antibody (145-2C11), FITC eBioscience 11-0031-82
CD44 Monoclonal Antibody (IM7), APC eBioscience 17-0441-82
Hamster Anti-Mouse CD69, PE BD Biosciences 553237
CD62L (L-Selectin) Monoclonal Antibody (MEL-14), PE eBioscience 12-0621-82
CD90.1 (Thy-1.1) Monoclonal Antibody (HIS51), FITC eBioscience 11-0900-81
CD103 (Integrin alpha E) Monoclonal Antibody (B-Ly7), APC eBioscience 17-1038-42
IFN gamma Monoclonal Antibody (XMG1.2), FITC eBioscience 11-7311-82
TNF alpha, PE, clone: MP6-XT22 eBioscience 501129406
KLRG1, APC, clone: 2F1 eBioscience 501123157
BV421 Rat Anti-Mouse CD127 Clone SB/199 BD Biosciences 562959
APC anti-mouse TNF-α Antibody Biolegend 506308
IL-17A Monoclonal Antibody (eBio17B7), PE eBioscience 12-7177-81
APC anti-T-bet Antibody Biolegend 644814

Validation

Data provided in the manuscript. Briefly, no customized antibodies were used. Validation data about the antibodies purchased from commercial vendors are available on the manufactures' website and in previous publications. We used primary cells or cell lines that are known to be positive for a specific antigen or target protein for validation of antibodies that were used for FACs and immunoblotting. Additionally, we chemically induced ER stress to validate the expression of the different ER stress and UPR-related proteins.

Eukaryotic cell lines

Policy information about cell lines

Cell line source(s)

293T cell line: 293T ATCC CRL-3216
B16 cell line: B16-F10 ATCC CRL-6475
EL-4 cell line: EL-4 ATCC TIB-39
3LL cell line: LL/2 (LLC1) ATCC CRL-1642
MCA-38 cell line: (MCA-38) Kerafast ENH204
ID8-Defb29/Vegf ovarian tumors Dr. Conejo- Garcia (Conejo-Garcia et al., 2004)

Authentication

Cell lines were not independently authenticated, beyond the identity provided from the supplier (e.g., ATCC).

Mycoplasma contamination

All cell lines were tested and negative for mycoplasma cotamination using an universal detection kit from ATCC.

Commonly misidentified lines
(See ICLAC register)

We did not use cell lines included in the ICLAC misidentified cell line database.

Animals and other organisms

Policy information about studies involving animals; ARRIVE guidelines recommended for reporting animal research

Laboratory animals

Mouse: Ddit3-/- (B6.129S(Cg)-Ddit3tm2.1Dron/J) Jackson Laboratory 005530
Mouse: Ddit3flox (B6.Cg-Ddit3tm1.1Irt/J) Dr. Ira Tabas N/A

	Mouse: Eif2ak3flox (Eif2ak3tm1.2Drc/J) Jackson Laboratory 005530<br>Mouse: CD4-cre<br>(B6.Cg-Thy1a/Cy Tg(TcraTcrb)8Rest/J) Jackson Laboratory 005023<br>Mouse: Pmel (B6.Cg-Tg(Cd4-cre)1Cwi/BfluJ) Jackson Laboratory 022071<br>Mouse: OT-1 (C57BL/6-Tg(TcraTcrb)1100Mjb/J) Jackson Laboratory 003831
Wild animals	The study did not involve wild animals.
Field-collected samples	The study did not involve samples collected from the field.
Ethics oversight	All animal studies are part of an approved IACUC protocol, reviewed by the Integrity and Compliance board at the University of South Florida and Moffitt Cancer Center.

Note that full information on the approval of the study protocol must also be provided in the manuscript.

Human research participants

Policy information about studies involving human research participants

Population characteristics	De-identified patients with advance ovarian carcinoma
Recruitment	De-identified patient's samples were available through an established bio-repository and a tissue micro-array prepared by the pathology core laboratory at Moffitt.
Ethics oversight	Experiments were covered through an approved IRB exemption protocol, reviewed by the Regulatory Affairs Committee Board at Moffitt Cancer Center.

Note that full information on the approval of the study protocol must also be provided in the manuscript.

Flow Cytometry

Plots

Confirm that:

☒ The axis labels state the marker and fluorochrome used (e.g. CD4-FITC).

☒ The axis scales are clearly visible. Include numbers along axes only for bottom left plot of group (a 'group' is an analysis of identical markers).

☒ All plots are contour plots with outliers or pseudocolor plots.

☒ A numerical value for number of cells or percentage (with statistics) is provided.

Methodology

Sample preparation	Digested tumor tissues or splenic cells were harvested and filtered, and then red blood cells lysed with ACK. For surface staining, cells were labelled with the appropriate antibodies in the presence of Fc blocker. For intracellular staining, surface-labeled cells were fixed with Cytofix/Cytoperm™ Solution (BD biosciences), washed in Permwash™ 1X solution, and labelled with intracellular antibodies. Cells were then washed in Permwash™ 1X and PBS and acquired by FACS. For the staining of Chop and T-bet, T cells were fixed with the Transcription Factor Fixation and Permeabilization kit (eBioscience), using the recommended protocol from the vendor. Cell live vs. dead discrimination was performed prior to antibody labeling by Zombie Violet™ Fixable Viability probe (BioLegend).
Instrument	CytoFLEX II, Beckman Coulter. In some experiments, samples were analyzed on an LSRII (BD) or FACSAria II unit (BD)
Software	FlowJo 10.3
Cell population abundance	The purity of the sorted cells was determined after FACS acquisition.
Gating strategy	Unless otherwise indicated, positive and negative gates were set using fluorophore-matched IgG controls. Please find details in the manuscript.

☒ Tick this box to confirm that a figure exemplifying the gating strategy is provided in the Supplementary Information.
